# First assessment of the health status of pregnant women, detection of prevalence and risk factors associated with enzootic ovine abortion disease among pregnant women in southern Benin

**DOI:** 10.3389/fpubh.2025.1532390

**Published:** 2025-05-21

**Authors:** Aboudou Habirou Kifouly, Ngemani Obase Bekindaka, Kaltun Said Ali, Juliana Rume, Michael Okunlola

**Affiliations:** ^1^Pan African University Life and Earth Sciences Institutes (Including Health and Agriculture), Ibadan, Oyo, Nigeria; ^2^Department of Obstetrics and Gynecology, University College Hospital, University of Ibadan, Ibadan, Oyo, Nigeria; ^3^Department of Animal Health and Production, University of Abomey-Calavi, Abomey-Calavi, Atlantique, Benin

**Keywords:** pregnant women, *Chlamydia abortus*, serology, prevalence, Benin

## Abstract

**Introduction:**

This study aims to access for the first time in the Benin Republic, the characteristics of the health status and the serological prevalence of *Chlamydia abortus* in pregnant women. Enzootic Abortion of Ewes (EAE) is a bacterial illness that can harm sheep by producing abortions and miscarriages in pregnant women.

**Methods:**

About six municipalities under two governorates were concerned and around 420 pregnant women were enrolled for the survey (210 participants in each governorate). Among of this enrollment, 385 participants were concerned for serological test.

**Results:**

Our result showed that this survey among pregnant women at the Sakété-Ifangni health zone hospital revealed that 125 participants (59.52%) had been exposed to potentially infected animals or products, with 40% having touched items from sick animals. Overall, 65.24% of animal owners were unsure whether they had been exposed. As much as 28 to 38% of the women farmed alongside their husbands, which frequently led to direct contact with aborted products. The consumption of milk from small ruminants was 26.67%. This consumption was associated with the risk of *Chlamydia abortus*. Half of them had experienced pregnancy complications. Knowledge of *Chlamydia abortus* varied from 16 to 68.5%. Proximity to small ruminant farms increases the risk of infection. Awareness among healthcare professionals needs to be improved. Although, the serological prevalence observed was relatively low (1.30%), it reveals a significant past exposure to the pathogen, especially in rural or cross-border areas such as the majority of the municipalities involved in this study.

**Conclusion:**

This data constitutes an epidemiology alert, justifying the introduction of additional methods such as PCR to access the active circulation and refine prevention strategies.

## Highlights

- The survey of pregnant women in our study revealed that tests for *Chlamydia abortus* infection are never administered, and worse still, other midwives and care assistants, as well as pregnant women, have never been aware of this infection.
- This observation was confirmed not only by the presence of numerous risk factors contributing to the suspicion of this disease in the environment of these pregnant women, but also by the serological analysis which revealed positive cases of *Chlamydia abortus* in the present study.
- Through this study, we suggested to the hospital administrative authorities that they require the *Chlamydia abortus* test during antenatal examinations.

## 1 Introduction

*Chlamydia abortus* (*C. abortus*) is a non-motile obligate intracellular Gram-negative pathogenic bacterium, belonging to the Chlamydiales family. *C. abortus* infects mainly ruminants, especially sheep and goats, and less frequently cattle, pigs and horses; however, it can also affect humans, being of particular concern in pregnant women (1, 2). *C. abortus* is known as the causative agent of enzootic abortion of ewes (EAE) or ovine enzootic abortion (OEA) which represents one of the most common causes of ovine and caprine infectious abortion worldwide, along with other infectious agents such as *Campylobacter* sp.*, Toxoplasma* sp.*, Listeria* sp.*, Salmonella* sp.*, Border disease virus* and *Cache Valley virus* (3, 4). Abortion occurs in the later stages of pregnancy, as *C. abortus* is able to progressively colonize the placenta, causing damage and affecting the fetus(es) to varying degrees (5). The infection can result in fetal loss (abortion), the birth of stillborn or weak lambs or, in some cases of unaffected animals; presence of a weak lamb with a healthy twin is also not uncommon (6). Breeders can incur great economic losses if numerous cases occur in a farm (abortion storm), usually when the infection first affects a naïve flock (7). Another important aspect is the spread of this enzootic infection to humans, which can develop as severe disease, especially in pregnant women (1), generally affecting female farmers, abattoir workers and veterinarians. However, environmental contamination with the bacteria released by abortion products or infected animals may also play a crucial role in disease spread, interspecies cross-over and adaptation (8). Indeed, abortion products, in particular vaginal fluids, placentas, dead/aborted lambs, fleeces and still born/infected lambs are all characterized by a high bacterial load and represent a significant risk, both for naïve animals and for humans (9). Infected and aborted animals shed the organism in placenta, uterine discharge, and feces, contaminating soils and generating infective aerosols that can be inhaled (9). Different types of flock management also influence the extent of environmental contamination and the spread of the pathogen. In intensively managed flocks, where animals are kept in smaller enclosures, there is a higher incidence of *C. abortus*, as the contamination is concentrated in confined spaces. Conversely, in extensively managed flocks, where animals are kept over larger areas, a lower incidence of the pathogen is observed, likely because animals are less exposed to contaminated environments (3). In addition, *C. abortus* can survive in the environment even in unfavorable conditions from a few days to a few months, thanks to the presence of a spore-like cell wall, which gives it considerable resilience (7). This resistance seems to be directly connected to the greater possibility for the bacterium to come into contact and infect many animal species, farmed or wild ones, and consequently to spread more easily to humans (1). Specific aspects of this will be discussed later.

The objective of this study was to assess for the first time, the characteristics of the health status and the serological prevalence of *C. abortus* in pregnant women in Benin, primarily in the governorate of Ouémé and Plateau.

## 2 Materials and methods

### 2.1 Period and type of study

This is a cross-sectional survey with a descriptive aim, conducted during the period from January to April 2023. This study followed the EQUATOR guidelines especially the extends STARD checklist.

### 2.2 Study area

The Ouémé department, with Porto-Novo as its capital, is characterized by its economic dynamism and fertile agricultural areas. The Plateau department, with its capital in Pobè, is distinguished by its hilly terrain, cultural richness, preservation of traditions, and traditional craftsmanship. Both regions encompass several municipalities, three (Ifangni, Sakété, and Adja-Ouèrè in Plateau department (Category 1), and Avrankou, Akpro-Missérété, and Dangbo in department of Ouémé; Category 2) of which have been included in this study. The Sakété-Ifangni health zone hospital, situated in Sakété within the Plateau region of Benin, serves as the focal point for this study.

### 2.3 Recruitment of participants

Pregnant women from prenatal clinics, health facilities, or maternity wards were recruited. This survey on enzootic sheep sickness was carried out from pregnant women attending prenatal clinics at the Sakété-Ifangni health zone hospital. These ladies were from diverse towns, the vast majority of which are within our research region. Avrankou, Akpro-Missérété, Dangbo, Adja-Ouèrè, Sakété, and Ifangni were among them.

### 2.4 Questionnaire

The questionnaire was created to gather information about the pregnant woman’s history of *C. abortus* infection, symptoms, risk factors, and sexual risk behaviors. The questionnaire also asked pregnant women about their knowledge and attitudes of *C. abortus* infection. The data were registered using KoboCollect. With that, more than 70 pregnant women were questioned by localities (06 localities in total) and around a total 420 were interrogated about their health status and their knowledge regarding *C. abortus* infections. The information was subdivided into two classifications for relevant comparisons. The category 1 is related to 3 municipalities of Plateau governorate which was: Ifangni, Sakété and Adja-Ouèrè and the second governorate were composed also 3 municipalities such as Avrankou, Akpro-Missérété and Dangbo.

### 2.5 Eligibility criteria

Inclusion criteria for pregnant women in a *Chlamydia abortus* study:

- Be pregnant, confirmed by ultrasound or a positive pregnancy test.
- Have a gestational age between 12 and 28 weeks.
- Be 18 years of age or older.
- Have given informed consent to participate in the study.
- Have regular access to health care during pregnancy.
- Pregnant women in the first trimester of pregnancy.

Exclusion criteria for pregnant women in a *Chlamydia abortus* study:

- Pregnant women in the second or third trimester of pregnancy
- Have been treated for chlamydia within the last 6 months.
- Have known HIV, hepatitis B, or hepatitis C infection.
- Have other sexually transmitted infections.
- Be on treatment for other chronic conditions that could affect pregnancy, such as diabetes or high blood pressure.
- History of pregnancy complications, such as pre-eclampsia or gestational diabetes
- Use of antibiotics in the 4 weeks before study inclusion
- Antibiotic use in the 30 days before study entry
- Diagnosis of *C. trachomatis* at the time of the study
- Alcohol or illicit drug use during pregnancy.

### 2.6 Sampling

The number of participants that were tested for *C. abortus* (via these clinical manifestations) were 385 pregnant women. Blood was drawn from the veins of the arm or wrist in dry tubes labeled with the patient’s medical record number. These tubes were then brought to the hospital laboratory and centrifuged at 3000 rpm for 5 min. This enabled us to separate the serum from the remainder of the blood and collect it in Eppendorf tubes labeled with the dry tube number.

### 2.7 Laboratory analysis

Diagnostic work has been carried out within the serology unit of the Bohicon Veterinary Laboratory. To test the sera, indirect ELISA kits provided by ID, VET companies (Montpellier, France) for *C. abortus* (chlamydiosis) were used. The tests were carried out following the manufacturer’s recommendations. Plate reading was performed using the ELISA reader (Chromate Inc. Germany) at 450 nm. For this zoonotic disease, the test was validated when the mean value of the optical density of the positive samples was greater than 0.350 and the ratio between the mean of the optical densities of the positive samples (ODpc) and those of the negative controls (ODnc) was greater than 3.

### 2.8 Statistical analysis

The data collected were analyzed to determine the prevalence of *C. abortus* infection, associated risk factors were calculated using the chi-square test, and knowledge and perceptions of infection by pregnant women were calculated using the student t-test.

## 3 Results

### 3.1 Distribution of pregnant women across different age

Table 1 shows the distribution of pregnant women across the different age groups, for both categories 1 and 2. In category 1, the largest number of pregnant women were in the 25–40 age group, accounting for 42.86% of the total. In category 2, the largest group of pregnant women were also in the 25–40 age group, representing 32.38% of the total. Notably, there is a significant difference in the percentages of pregnant women between the two categories in these age groups, with a *p*-value of 0.004, indicating a statistically significant difference between the two categories.

**Table 1:** Distribution of pregnant women across different age.

Age groups	Category 1 (210)			Category 2 (210)			*p* value
	No. of examined pregnant women	%	95% CI	No. of examined pregnant women	%	95% CI	
18–20	26	12.38	0.95 ± 27.18	27	12.86	1.55 ± 28.50	0.004*
20–25	52	24.76	26.32 ± 48.68	73	34.76	28.59 ± 51.21	
25–40	90	42.86	17.261 ± 41.07	68	32.38	18.04 ± 42.06	
40-à plus	42	20.00	6.56 ± 31.98	42	20.00	3.51 ± 26.54	
Total	210	100.00	0.83 ± 3.85	210	100.00		

*The result of student t test is significant at *p* < 0.05; and non-significant at *p* > 0.05.

### 3.2 Factors influencing pregnancy risks among pregnant women

Table 2 presents on Factors Influencing Pregnancy Risks among pregnant women. Significant correlations were observed between the factors (such as indirect exposure to sick animals, housing type, farmer classification, previous illnesses, and the presence of sick animal products) and pregnancy risks among pregnant women. Indirect contact with sick animals was significantly more prevalent in category 2 (73.3%) compared to category 1 (59.5%; *p* = 0.000). Similarly, there was a higher incidence of indirect exposure to infected animal products in category 2 (47.1%) than in category 1 (40.5%), though not statistically significant (*p* = 0.053). Additionally, hygiene practices, such as disinfection after contact, were significantly less common in category 2 (18.1%) than in category 1 (24.3%; *p* = 0.005). Living near a livestock farm was also significantly less frequent in category 2 (31.9%) than in category 1 (39.1%; *p* = 0.026). Furthermore, mixed breeding’s were significantly more prevalent in category 2 (37.6%) than in category 1 (27.6%; *p* = 0.006). Underlying diseases were significantly less common in category 2 (33.3%) compared to category 1 (40.0%; *p* = 0.040). In summary, these findings suggested riskier exposures but better hygiene practices in category 1, while category 2 appears to have overall better health outcomes, indicating the necessity for targeted preventive measures to combat a likely incidence of *C. abortus* infection.

**Table 2:** Factors influencing pregnancy risks among pregnant women.

Parameters	Category 1 (210)			Category 2 (210)			*p* value
	No. of examined pregnant women	%	95% CI	No. of examined pregnant women	%	95% CI	
Indirect contact with sick animals							
Yes	125	59.52	54.19 ± 70.25	154	73.33	63.12 ± 78.21	0.000*
No	85	40.48	27.47 ± 48.08	56	26.67	19.65 ± 39.01	
	210			210			
Indirect contact with sicks animals’ products							
Yes	85	40.48	31.88 ± 48.12	99	47.14	38.40 ± 54.93	0.053*
No	125	59.52	49.59 ± 70.41	111	52.86	42.73 ± 63.94	
	210			210			
Hygiene after contact							
Disinfection	51	24.29	16.77 ± 31.80	38	18.10	11.35 ± 24.84	0.005*
Washing with water	96	45.71	35.12 ± 56.30	88	41.90	31.42 ± 52.39	
Washing with soap	63	30.00	23.80 ± 36.20	84	40.00	33.37 ± 46.63	
	210			210			
Housing							
Next to livestock farms	82	39.05	28.47 ± 44.42	67	31.90	23.73 ± 40.08	0.026*
In built-up areas	128	60.95	46.36 ± 67.42	143	68.10	58.19 ± 78.00	
	210			210			
Type of farmers							
Men	69	32.86	24.62 ± 41.09	65	30.95	22.85 ± 39.06	0.006*
Women	83	39.52	29.13 ± 49.92	66	31.43	21.56 ± 41.30	
Mixed	58	27.62	21.57 ± 33.67	79	37.62	31.07 ± 44.17	
	210			210			
Previous diseases							
Underlying illness	84	40.00	31.41 ± 48.59	70	33.33	25.07 ± 41.60	0.040*
Other medical condition	126	60.00	49.59 ± 70.41	140	66.67	56.64 ± 76.69	
	210			210			

*The result of student t test is significant at *p* < 0.05; and non-significant at *p* > 0.05.

### 3.3 Factors influencing awareness of *Chlamydia abortus* on pregnancy

Table 3 compares categories on zoonotic disease prevention (*C. abortus*) in pregnant women. Preventive measures against zoonoses, included *C. abortus*, were significantly more prevalent in category 2 (62.9%) than in category 1 (54.8%; *p* = 0.015). Similarly, knowledge of available treatments for *C. abortus* was significantly higher in category 2 (64.3%) than in category 1 (56.2%; *p* = 0.014). History of genitourinary infections was also significantly more common in category 2 (58.1%) than in category 1 (50.5%; *p* = 0.025). Obstetric complications were more frequent in category 2 (59.1%) than in category 1 (51.9%; *p* = 0.035). However, knowledge of *C. abortus* and its associated risks was lower in category 2 (39.1%) compared to category 1 (46.2%), significantly (*p* = 0.034). In summary, despite improvements in prevention and clinical management, *C. abortus* infection possibly remained a concern in category 2, emphasized the need for enhanced education and screening.

**Table 3:** Factors influencing awareness of *C. abortus* on pregnancy complications among the pregnant women examined.

	Category 1 (210)			Category 2 (210)			
Parameters	No. of examined pregnant women	%	95% CI	No. of examined pregnant women	%	95% CI	*p* value
Preventive measures to avoid zoonotic diseases. Including *Chlamydia abortus*							
Yes	115	54.76	46.04 ± 63.49	132	62.86	54.39 ± 71.33	0.015*
No	95	45.24	34.66 ± 55.82	78	37.14	26.87 ± 47.42	
	210			210			
Awareness of available treatment of *C. abortus*							
Yes	118	56.19	47.49 ± 64.89	135	64.29	55.89 ± 72.69	0.014*
No	92	43.81	33.26 ± 54.36	75	35.71	25.53 ± 45.90	
	210			210			
History of genital or urinary tract infection							
Yes	106	50.48	41.71 ± 59.24	122	58.10	49.45 ± 66.74	0.025*
No	104	49.52	38.89 ± 60.15	88	41.90	31.42 ± 52.39	
	210			210			
History of pregnancy complication							
Yes	109	51.90	43.15 ± 60.66	124	59.05	50.43 ± 67.67	0.035*
No	101	48.10	37.47 ± 58.72	86	40.95	30.50 ± 51.41	
	210			210			
Complication about pregnancy							
Yes	125	59.52	50.92 ± 68.13	110	52.38	43.63 ± 61.14	0.038*
No	85	40.48	30.04 ± 50.91	100	47.62	37.00 ± 58.24	
	210			210			
Heard of *C. abortus* and risks							
Yes	97	46.19	37.45 ± 54.93	82	39.05	30.50 ± 47.60	0.034*
No	113	53.81	43.21 ± 64.41	128	60.95	50.58 ± 71.32	
	210			210			

*The result of student t test is significant at *p* < 0.05; and non-significant at *p* > 0.05.

### 3.4 Associated risks factors of *Chlamydia abortus* among pregnant women

Table 4 shows pregnant women differences in interactions, farm proximity, milk consumption, risk awareness, and symptoms. Significant *p*-values indicate impact on outcomes of pregnancy. Women who frequently visited small ruminant farms were significantly more prevalent in category 1 (35.7%) than in category 2 (33.3%; *p* = 0.013). Similarly, women living within 5 km of these farms were significantly more prevalent in category 2 (39.5%) than in category 1 (30.0%; *p* = 0.019). Regular consumption of small ruminant milk was also significantly higher in category 2 (23.8%) than in category 1 (14.8%; *p* = 0.009). Potential exposure to infected animals was reported by 34.8% of women in category 1 compared to 28.1% in category 2, significantly (*p* = 0.032). However, awareness of the risk of *C. abortus* infection was significantly higher in category 2 (41.9%) than in category 1 (34.8%; *p* = 0.04). Additionally, a history of abortions was significantly more common in category 2 (57.6%) than in category 1 (51.4%; *p* = 0.036). In summary, certain interactions with small ruminants seemed to elevate the risk of *C. abortus* infection in pregnant women, underscoring the need for improved education and preventive healthcare measures.

**Table 4:** Associated risks factors of *C. abortus* among pregnant women.

	Category 1 (210)			Category 2 (210)			
Parameters	No. of examined pregnant women	%	95% CI	No. of examined pregnant women	%	95% CI	*p* value
Visit of small ruminant farms							
Frequently	75	35.71	27.31 ± 44.11	70	33.33	25.07 ± 41.60	0.013*
Often	115	54.76	44.18 ± 65.34	104	49.52	38.89 ± 60.15	
None	20	9.52	5.55 ± 13.49	36	17.14	12.05 ± 22.24	
	210			210			
Live nearest of small ruminant farms							
Around 5 km	87	41.43	32.79 ± 50.06	75	35.71	27.31 ± 44.11	0.019*
Less than 5 km	63	30.00	20.26 ± 39.74	83	39.52	29.13 ± 49.92	
More than 5 km	60	28.57	22.46 ± 34.68	52	24.76	18.92 ± 30.60	
	210			210			
Consumption of milk from small ruminant							
Never	123	58.57	49.94 ± 67.21	111	52.86	44.11 ± 61.61	0.009*
Often	56	26.67	17.27 ± 36.07	49	23.33	14.34 ± 32.32	
Always	31	14.76	9.96 ± 19.56	50	23.81	18.05 ± 29.57	
	210			210			
Exposition to animal infected							
Unknow	137	65.24	56.89 ± 73.59	151	71.90	64.03 ± 79.78	0.032*
Perhaps	73	34.76	24.64 ± 44.89	59	28.10	18.54 ± 37.65	
	210			210			
Informed about potential risk of *C. abortus*							
Yes	73	34.76	26.41 ± 43.11	88	41.905	33.26 ± 50.55	0.04*
No	137	65.24	55.11 ± 75.36	122	58.095	47.61 ± 68.58	
	210			210			
Experienced of disease symptoms							
Abortion	108	51.43	42.67 ± 60.19	121	57.62	48.96 ± 66.28	0.036*
Miscarriage	79	37.62	27.32 ± 47.92	62	29.52	19.83 ± 39.22	
Stillbirth	23	10.95	6.73 ± 15.18	27	12.86	8.33 ± 17.38	
	210			210			

*The result of student t test is significant at *p* < 0.05; and non-significant at *p* > 0.05.

### 3.5 Prevalence of *Chlamydia abortus*

Table 5 shows the prevalence of *C. abortus* in pregnant women in different regions. The overall incidence was relatively low, with only 5 positive cases out of a total of 385 women tested (1.3%). Ifangni and Sakété municipalities had the highest incidences, at 1.49 and 2.67%, respectively. Conversely, Adja-Ouèrè and Dangbo municipalities have reported no positive cases. Despite the low seroprevalence, it underscored the presence of *C. abortus* among the demographic of pregnant women in these regions.

**Table 5:** Prevalence of *C. abortus* cases among expectant women.

Municipalities	No. of examined pregnant	No. of positive	%	95% CI	*p* value
Ifangni	67	1	1.49	−1.41 ± 4.40	6.89E-79*
Sakété	75	2	2.67	−0.98 ± 6.31	
Adja-Ouèrè	50	0	0.00	0	
Avrankou	67	1	1.49	−1.41 ± 4.40	
Dangbo	48	0	0.00	0	
Akpro-Missérété	78	1	1.28	−1.21 ± 3.78	
Total	385	5			

*The result of student t test is significant at *p* < 0.05; and non-significant at *p* > 0.05.

## 4 Discussion

The survey of pregnant women attending an antenatal clinic for the first time highlights the proportion of pregnant women surveyed who had been exposed to animals suspected of being infected or to potentially contaminated animal products such as milk or blood in the course of their work or daily activities. The results show that more than half of the pregnant women surveyed (59.52%) had been exposed to sick animals indirectly, and almost half of these women (40%) said that they had at least touched sick animal products (blood, milk, saliva, snot, etc.) indirectly as part of their work or daily activities. This observation can be justified by the fact that nearly 40% of the pregnant women surveyed were livestock owners and that the majority of them (65.24% and 72% in both categories) did not know whether or not they were exposed to infected animals; while nearly 28% and 38% of these women also practiced animal husbandry jointly with their husbands, and because of the proximity, nearly 52% and 58% in both categories stated that they often touched aborted products. The prevalence of women reporting contact with diseased or dead animal products is relatively higher than that reported by (10), who found that 26% of women interviewed in their study had been in contact with dead animal products. However, regarding the consumption of small ruminant milk, the results presented indicate that only 26.67% of the women surveyed responded that they often consumed it. However, this proportion may be underestimated as a large number of women did not answer this question. Nevertheless, this prevalence is lower than that reported by (11), who found that 29.8% of the women surveyed in their study had consumed goat’s or sheep’s milk. In addition, a study by (12) in Germany showed that consumption of raw goat’s or sheep’s milk was a significant risk factor for *C. abortus* infection in pregnant women. This study suggests that the consumption of milk from small ruminants may play an important role in the transmission of *C. abortus* to pregnant women.

Interesting results on the medical history of the pregnant women interviewed with regard to *C. abortus* were collected during this survey. It is of concern that some of the women surveyed had a history of genital or urinary tract infections, as this may increase the risk of infection during pregnancy. In terms of symptoms, it was found that almost half of the women had experienced abortions, miscarriages and stillbirths in both departments during their maternity career. Our results are similar to those obtained by (13) who showed that 25% of pregnant women had a *C. abortus* infection, but lower than those found by (14) who showed that 43.3% of pregnant women infected with *C. abortus* presented symptoms such as vaginal discharge, abdominal pain or vaginal bleeding. These symptoms may be associated with *C. abortus* infection, but it is important to note that they may also be caused by other infections or pathologies. Finally, a study by (5) showed that a history of *C. abortus* infection was associated with an increased risk of complications during pregnancy, such as spontaneous abortion or preterm labor. The findings were concerning, revealing that half of the women had experienced pregnancy complications potentially linked to infection, while most lacked adequate awareness of Chlamydia abortus during pregnancy.

These results are in agreement with several other studies conducted on this subject. A study conducted by (15) found that only 16% of pregnant women surveyed were aware of the possibility of transmission of *C. abortus* from mother to child during pregnancy. Similarly, a study by (16) found that only 28% of pregnant women surveyed were aware of *C. abortus* and its consequences for the health of mother and child. However, other studies have reported higher rates of knowledge than the 20.52% found. For example, a study by (17) found that 67.8% of pregnant women surveyed were aware of *C. abortus*. Similarly, a study by (18) showed that 68.5% of pregnant women surveyed were aware of *Chlamydia* abortus and its impact on the health of mother and child.

Interesting information on the proximity and frequency of visits by pregnant women to small ruminant farms has been collected. However, a study by (19) revealed that small ruminants are important reservoirs of *C. abortus*, which can be transmitted to pregnant women through direct or indirect contact. The results of the present study are consistent with this observation, as more than half of the women surveyed responded that they lived near small ruminant farms. In addition, another study by (20) also examined the prevalence of *C. abortus* in small ruminants in Hungary. The authors found that the prevalence of infection was higher in small ruminant farms located in rural areas, which is consistent with the results presented in our study. In addition, a study by (1) evaluated the efficacy of different vaccines against *C. abortus* in small ruminants. The authors emphasized the need to control infections in small ruminants to prevent zoonotic transmission. Accordingly, raising awareness among pregnant women about the risks linked to close contact and regular visits to small ruminant farms is crucial. The results mentioned also that the majority of pregnant women are not well informed about the risks and means of preventing *C. abortus* during pregnancy, or about the advice given by healthcare professionals. These results are in line with other studies conducted on the subject. A study conducted by (21) among pregnant women in an antenatal clinic in Italy showed that only 33.5% of women knew about *C. abortus* and its risks during pregnancy. Similarly, a study conducted by (22) in the UK showed that only 25.6% of pregnant women were aware of *C. abortus*. In addition, these findings also highlight the need to improve knowledge of *C. abortus* among healthcare professionals, so that they can provide adequate advice and information to pregnant women. Previous studies have also highlighted the need for adequate training of healthcare professionals on awareness, screening and treatment of *C. abortus* during pregnancy (21), (22).

In addition, the study found a serological prevalence of 1.30%, i.e. five pregnant women out of the 385 examined, with positive results. These women were isolated for observation, revealing the presence of symptoms previously listed as well as a clinical history related to sexually transmitted infections and other genital infections. These observations corroborate previous findings by (23) and (24), both of whom identified this pathogen. Ortega identified it in a woman working in a laboratory, while Pichon linked it to a woman presenting with pelvic pain during her ante-natal consultation. This study highlights the urgent need to incorporate medical examinations targeting *C. abortus* into antenatal consultations for pregnant women in hospitals in the health zones of the Ouémé and Plateau governorates.

## 5 Conclusion

This study provides the first serological assessment of *Chlamydia abortus* among pregnant women in Benin. Despite a low prevalence (1.30%), the high exposure rates and associated risk factors highlight significant public health concerns. These findings call for improved awareness, targeted surveillance using molecular tools, and enhanced preventive strategies, especially in rural and cross-border areas.

## Data Availability

The raw data supporting the conclusions of this article will be made available by the authors, without undue reservation.
